# Homœopathy Legitimately Developed

**Published:** 1855-03

**Authors:** 


### HOMŒOPATHY LEGITIMATELY DEVELOPED.

The New York Medical Journals, make lengthy allusions to a recent case of death by homœopathy, which has lately occurred in Brooklyn. The particulars involve such a stupendous amount of ignorance and obstinacy in the attending physicians, as to stir to indignation the sensibilities of any man. We have only room to mention, very briefly, a bare outline of the facts.

A pamphlet has been published, containing the history of the case, together with the inquest before the coroner, and the strange testimony of the Homœopathic Doctors. It seems that a little girl, Agnes P. Lottimer, of Brooklyn, was taken ill, August 2nd; and a Dr. Wells, having been called in, found as he alleges inflammation of the brain;—with all the accompanying symptoms, although other circumstances and the progress of the case, proved plainly enough that she was in the hot stage of an intermittent. The Dr. goes on to treat his mistaken cerebral disease with miraculously small doses of aconite and belladonna. We are not in the mood for nice calculations. The quantity given, was the merest shadow of a dilution of the tinctures of aconite and belladonna in some millions of gallons of water; just how many millions, is a a matter of no consequence, in a homœopathic point of view. Under this treatment, the "inflammation of the brain," at which our Dr. Wells was insanely aiming his sugar water, did not subside. The microscopic treatment went on, and so did the fever and ague. Rhus, toxicodendron, pulsatilla were called in use, but did no good. Week after week progressed, and the disease unchecked, kept pace with time. Chill followed chill, with alarming and fearful regularity, and with increasing violence; the little sufferer, as appears from the testimony, was "pale; extremities cold; the whole body covered with a clammy sweat; thirst almost continual; tossing of the body to and fro; anxious sighing respiration; heat of the head to the touch; pulse very frequent and very feeble." Dr. Dunham was called in, and illuminated the case by additional homeopathic darkness. We cannot follow the painful progress of the case. It went on for *more* than two months, the disease becoming of course more and more complicated, and as it advanced, fastening itself upon fresh structures. At last, the patient was seized with convulsions, and finally with hemorrhage from the lungs, which soon proved fatal. All this time, and even up to the very close, the parents were consolingly assured by the doctor that their child was improving.

Now, the evidence before the coroner, clearly proved, that this child died of fever and ague, and that too of a type of no uncommon severity. The disease, it is proved, was allowed to run its course unchecked, and death ensued. Look at the case. Everybody, educated or not, knows that thousands and tens of thousands of cases of fever and ague are cured every year, in this country, by physicians and even ignorant people, sometimes, by the simple use of a few grains of calomel or blue pill, castor oil, and by quinine. It is as plain and clear as that the sun rises every morning. Yet, in the city of Brooklyn, a child is taken sick with ordinary intermittent, a doctor is called in, who at once sees the nature of the case, (if not, so much the worse,) and yet, knowing the entire curability of the disease, but by means, which his *creed*, does not acknowledge, he is so wedded to his insane fooleries, that he sits by, and sees her go down and down, knows, or should know that fearful congestions are accumulating in the system, and for two months allows her to struggle unaided with the disease, and finally sees her die, without once stretching forth his hand, or making one intelligent effort to save her. The patient must die before he will stir from his homeopathic orthodoxy. It is but the very tamest language to say, that such a course of conduct is only a formal round-about homicide, and that any doctor thus guilty, should be held as criminal, in the eyes of the law.

If anything can add to the revoltingly criminal aspect of this painful narrative, look at the profoundly deliberate ignorance of the homœopathic doctors, as evinced by their testimony before the coroner's jury. The statement was brought forward by these savans, that the child died of—what would you suppose?—*mumps!* The allegation was, that three days before her death, the disease became complicated with mumps, which was translated to the membranes of the brain. It has been a notion that mumps generally involve a certain gland, sometimes called the parotid; but these mumps never affected that organ. What were the symptoms? "A slight stiffness of one side of the neck, with slight tenderness on pressure over the upper and anterior edge of the cleido-mastoid muscle. *There was no swelling of the parotid gland.*" Remarkable mumps, indeed! yet not more so than mumps in the practice of doctor Hull, another such, who testified of a child who had mumps in its bowels! Well, in this case, as the child died of *mumps in the brain*, it seemed necessary to account for the fatal hœmorrhage from the same source; and how was that done? Our Dr. Wells shows his contempt of pathology by alleging that she died "by suffocation from the accumulation of blood in the throat," said blood having found its way there out of the longitudinal sinus, which, having been ruptured, poured its contents *through the cribriform plate of the ethmoid bone!*

Mumps, without any affection of the parotid gland, and such mumps translated to the brain, then to the lungs, and finally fatal hemorrhage through the ethmoid bone!! Pathology is no impediment to the progress of such a man.

A cotemporary says: "The amount of moral guilt involved in such cases we will not presume to determine. Yet it is plain enough that to mean well is not a sufficient reason for doing ill, nor to think one's self right, a proper excuse for doing wrong." We heartily endorse it, with an earnest affirmation on our part respecting the criminality of ignorance, when plainly voluntary, as here. Such ignorance is doubly culpable. The individual is in the first place deeply to blame for his ignorance, and also for that wanton abuse of life, which is the result of such ignorance. We have no patience with smooth-faced pretenders. We only regret that our want of space prevents any further comments.

